# Quantification of spatial-temporal light interception of crops in different configurations of soybean-maize strip intercropping

**DOI:** 10.3389/fpls.2024.1376687

**Published:** 2024-09-30

**Authors:** Fu Jin, Zhihua Wang, Haizhao Zhang, Sirong Huang, Meng Chen, Titriku John Kwame, Taiwen Yong, Xiaochun Wang, Feng Yang, Jiang Liu, Liang Yu, Tian Pu, Akash Fatima, Raheela Rahman, Yanhong Yan, Wenyu Yang, Yushan Wu

**Affiliations:** ^1^ College of Agronomy, Sichuan Agricultural University, Chengdu, China; ^2^ Sichuan Engineering Research Center for Crop Strip Intercropping System, Sichuan Agricultural University, Chengdu, China; ^3^ Key Laboratory of Crop Eco- physiology and Farming System in Southwest of China, Sichuan Agricultural University, Chengdu, China; ^4^ Institute of Plant Breeding and Biotechnology, Muhammad Nawaz Shareef University of Agriculture, Multan, Pakistan; ^5^ Department of Plant Breeding and Genetic, University of Agriculture, Faisalabad, Pakistan; ^6^ College of Grassland science and technology, Sichuan Agricultural University, Chengdu, China

**Keywords:** intercropping, light interception, model, soybean, maize

## Abstract

Intercropping can improve light interception and crop yield on limited farmlands. The light interception rate in intercropping is determined by row configuration. Quantifying the spatio-temporal light interception of intercrops is very important for improving crop yields by optimizing the row configuration. A two-year field experiment was conducted at two sites to quantify the responses of the light interception rate of intercrops to five treatments: two rows of maize alternated with three rows of soybean (2M3S), two rows of maize alternated four rows of soybean (2M4S), two rows of maize alternated five rows of soybean (2M5S), sole soybean (SS), and sole maize (SM). We developed a multiple regression model based on the sine of the solar elevation angle (sin(h)) and crop leaf area density (LAD) to quantify the spatio-temporal light interception of intercrops. The predicted light interception rate was positively correlated with the measured values of photosynthetically active radiation (R^2^ > 0.814) and dry matter (R^2^ > 0.830). Increasing soybean rows led to an increase in light interception of both soybean and the lower layer of maize. However, this also resulted in a decrease in light interception in the upper layer of maize. At the two sites, compared to 2M3S, the annual average cumulative light interception of soybean in 2M5S increased by 44.73% and 47.18%, that of the lower layer of maize in 2M5S increased by 9.25% and 8.04%, and that of whole canopy of maize decreased by 13.77% and 17.74% respectively. The changes in dry matter and yield of intercrops were consistent with the change in light interception, which further verified the high accuracy of the light interception model. The annual average maize yield of 2M5S was 6.03% and 6.16% lower but the soybean yield was 23.69% and 28.52% higher than that of 2M3S. On the basis of system yield, the best performance was recorded in 2M4S at the two sites. In summary, the newly created light interception model performs well in the quantification of the temporal and spatial changes in crop light interception in strip intercropping and has potential applications in other configurations. Optimizing row configurations across climatic regions to enhance light interception and yield at the system level will become a future target.

## 1 Introduction

In recent years, global food security has become vulnerable to an increase in population, decrease in arable land, climate anomalies, conflicts, and economic downturns (Du et al., 2018; Boliko, 2019; Molotoks et al., 2021). Intercropping is considered a promising planting pattern to ensure food security because of its ability to achieve high and stable yields (Stomph et al., 2020; Liu et al., 2023). Intercropping refers to the simultaneous cultivation of two crops on the same land area. It can not only improve agricultural production but also achieve economic and environmental benefits through the diversification of crop combinations (Waha et al., 2020; Zou et al., 2021; Huss et al., 2022). Cereal-legume intercropping is an excellent method for improving efficiency, productivity and sustainability (Wu et al., 2014; Du et al., 2018; Dai et al., 2019; Iqbal et al., 2019; Raza et al., 2021).

Crop light interception has a greater effect on yield (Stewart et al., 2003; Edwards et al., 2005). Accurate quantification of the light interception of intercropped crops is essential to evaluate the yield formation process. Light interception models are crucial for understanding and optimizing intercropping systems, which involve growing multiple crops in close proximity. Multilayer light interception models (2D) have been developed to estimate light interception in heterogeneous canopies based on Beer’s law, which divides the crop canopy into many layers in the canopy vertical direction (Sinoquet and Bonhomme, 1991; 1992). There are two types of multilayer light interception models: one based on radiative transfer and the other based on a simple statistical approach (Wang et al., 2015, 2021a).

The radiative transfer model incorporates various parameters, such as crop Leaf area index (LAI), leaf distribution, and canopy characteristics to account for the different pathways through which light travels within the canopy (Wang et al., 2017). The model was shown to provide more precise simulation results for intercropping systems. For instance, Munz et al. (2014) focused on the daily variation in light intensity at the top of each row of common bean (*Phaseolus vulgaris L.* var. *nana*) in a strip intercropping system with maize. Wang et al. (2017) further enhanced the accuracy of the light transmission model by refining it to predict the instantaneous light interception of each intercrop row in a maize and wheat (*Triticum durum*) strip intercropping system. Liu et al. (2022) investigated how the variations in strip width and row orientation affect light interception in a maize and soybean strip intercropping system. The radiative transfer model has been extensively applied to study light interception in various intercropping systems, including maize/wheat, maize/soybean, wild pea (*genus Vicia*)/oat (*Avena sativa L.*), and maize/peanut (*Arachis hypogaea L.*) strip intercropping, and reliable results have been obtained (Wang et al., 2015; Liu et al., 2017a, b; Li et al., 2021a; Wang et al., 2021b). Furthermore, some researchers have documented a simplified statistical method for assessing the light interception in intercropping systems. Qi et al. (2021) focused on the disparity in light intensity between the top and bottom of the crop canopy to define light interception. Wang et al. (2021a) quantified light interception in intercropped systems by multiplying the intercrop’s footprint with the light interception of a single crop. Rathika et al. (2013) took a different approach, considering the ratio between the difference in light intensity at the top and bottom of the canopy and the intensity at the top to evaluate light interception. Nevertheless, it is important to note that these light-interception algorithms, which rely on limited data, may not accurately capture the unique benefits of supplemental light in the edge rows of strip intercropping. Furthermore, the primary focus of these studies was to understand the daily variation in light interception. Spatio-temporal heterogeneity within intercropping systems poses a challenge in accurately estimating crop light interception. Although the aforementioned light interception models have significantly improved the efficiency of estimating light interception in intercropping systems, they have certain limitations. These models overlook intricate processes related to light distribution and transmission within the crop canopy. Second, they lack the ability to compute light interception on smaller temporal and spatial scales. Consequently, these models fail to comprehensively and realistically reflect the spatio-temporal light interception of intercropped crops.

To improve the adaptability and realism of the light interception model for strip intercropping, it is essential to incorporate actual measured data into its development process (Orlov et al., 2021; Zhao et al., 2021). It has the advantages of accurate calculation results, ease of use, and stability (Jaswon, 1963). The data-driven type of light interception model refers to a model that can accurately evaluate light interception, crop growth, and yield in monocrops (Bulgakov et al., 2015; Xue et al., 2015; Bai et al., 2016; Malladi and Sowlati, 2018; Sadenova et al., 2021), and is based on actual measurement data of light intensity from the field and numerical integration. However, studies on intercrops have not yet been reported in the literature.

In this study, we focused on maize-soybean strip intercropping with the aim of (1) developing a novel spatio-temporal light interception model with fewer parameters to quantitatively assess light interception in different configurations and regions, and (2) identifying the optimal row configuration by quantifying crop light interception and yield.

## 2 Materials and methods

### 2.1 Experimental site

**Figure 1:**
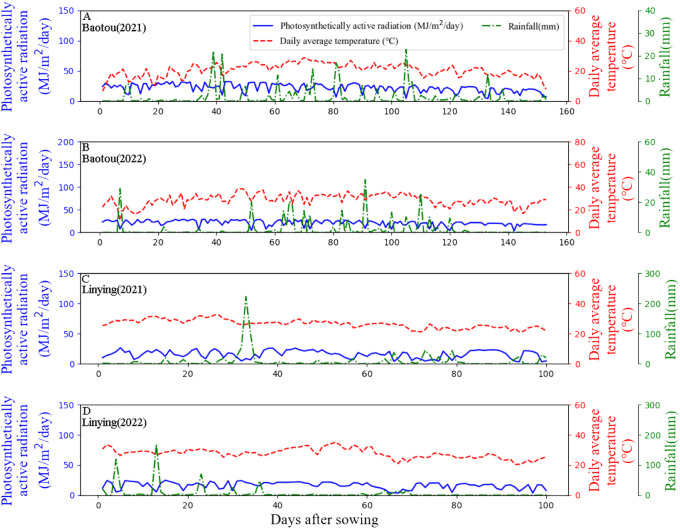
Meteorological data for 2021 and 2022 at the Baotou and Linying experimental sites. **(A, B)** represent the Baotou site in 2021 and 2022. **(C, D)** represent the Linying site in 2021 and 2022.

The field experiment was carried out in 2021-2022 in Baotou City, Inner Mongolia (N40°35′5.53″, E110°28′59.35″), and Linying City, Henan (N33°46′13.79″, E113°50′26.98″), China. Baotou has a typical continental semi-arid monsoon climate, with an average annual air temperature of 7.5°C, 135 frost-free days and 3095 sunshine hours per year. The mean annual rainfall was 346 mm. Linying has a temperate monsoonal climate. The mean annual air temperature was 14.5°C, and there were 226 frost-free days. The average annual rainfall was 720 mm. The soil at the two experimental sites is clay. Meteorological data obtained during the experiment are shown in **Figure 1**.

### 2.2 Experimental design

The field study had a completely randomized block design with three replicates. There were five planting patterns: sole maize (SM), sole soybean (SS), two rows of maize alternating with three rows of soybean (2M3S), four rows of soybean (2M4S), and five rows of soybean (2M5S). The detailed field configuration parameters are listed in (**Table 1**). All strips were oriented east-west. At the Baotou site, the maize cultivar was *Denghai618* with a density of 75,000 plants ha^-1^ for sole and intercropping, and the soybean cultivars were *Zhonghuang30* in 2021 and *Jiyu86* in 2022, with densities of 225,000 plants ha^-1^ and 150,000 plants ha^-1^ for sole and intercropping, respectively. Both soybean and maize were sown on May 1, 2021, and May 1, 2022, and harvested at October 1, 2021, and October 1, 2022. At the Linying site, the maize cultivar was *Zhengdan958* with a density of 67,500 plants ha^-1^ for sole and intercropping, the soybean cultivar was *Qihuang34* with a density of 225,000 plants ha^-1^ for sole and 150,000 plants ha^-1^ for intercropping; both soybean and maize were sown on June 18, 2021, and June 22, 2022, and harvested on October 7, 2021, and October 4, 2022.

**Table 1:** The field configurations of maize soybean strip intercropping.

Site	Treatment	Strip width (cm)	Distance between maize and soybean (cm)	Maize			Soybean		
				Density (plants ha^-1^)	Row spacing (cm)	Plant spacing (cm)	Density (plants ha^-1^)	Row spacing (cm)	Plant spacing (cm)
Baotou	SS	–	–	–	–	–	225,000	50	8.9
	SM	–	–	75,000	70	19.0	–	–	–
	2M3S	220	60	75,000	40	12.1	150,000	30	9.1
	2M4S	250	60	75,000	40	10.7	150,000	30	10.7
	2M5S	280	60	75,000	40	9.5	150,000	30	11.9
Linying	SS	–	–	–	–	–	225,000	50	8.9
	SM	–	–	67,500	70	21.2	–	–	–
	2M3S	220	60	67,500	40	13.5	150,000	30	9.1
	2M4S	250	60	67,500	40	11.9	150,000	30	10.7
	2M5S	280	60	67,500	40	10.6	150,000	30	11.9

SS represents sole soybean with a density of 225,000 plants ha^-1^at the Baotou and Linying sites, SM represents sole maize with a density of 75,000 plants ha^-1^ at the Baotou site and 67,500 plants ha^-1^ at the Linying site; 2M3S represents two rows of maize alternated with three soybean rows with a maize density of 75,000 plants ha^-1^ at the Baotou site and 67,500 plants ha^-1^ at the Linying site; 2M4S represents two rows of maize alternated with four soybean rows with a maize density of 75,000 plants ha^-1^ at the Baotou site and 67,500 plants ha^-1^ at the Linying site; 2M5S represents two rows of maize alternated with five soybean rows with a maize density of 75,000 plants ha^-1^ at the Baotou site and 67,500 plants ha^-1^ at the Linying site.

Fertilizer was applied according to the planting density and was held at 10 cm near the crops. At the Baotou site, based on local maize production, 364 kg N ha^-1^ was supplied to the sole maize with a planting density of 75, 000 plants ha^-1^. The N for maize was divided into two parts: 157 kg N ha^-1^ was applied as a base fertilizer, and 207 kg N ha^-1^ was applied as a topdressing at the maize tassel stage. A base fertilizer of 225 kg N ha^-1^ was applied to the soybean. At the Linying site, based on the local maize production, 270 kg N ha^-1^ was supplied to the sole maize with a planting density of 67, 500 plants ha^-1^. The N for maize was divided into two parts, 127 kg N ha^-1^ was applied as base fertilizer, and 143 kg N ha^-1^ was applied as topdressing at the maize tassel stage. A base fertilizer of 60 kg N ha^-1^ was applied to the soybean. Weeds, insect pests, and diseases were properly controlled and crops were managed so that they were not limited by other nutrients. Sprinkler irrigation was used at the Linying site, while drip irrigation was used at the Baotou site. Both irrigation methods were in line with the local production practices. Water was provided separately to meet crop growth requirements, especially during the critical reproductive periods.

### 2.3 Data collection

#### 2.3.1 Crop morphology and dry matter

At the Baotou site, the height and leaf area of maize and soybean were measured 29, 44, 62, 81, and 119 days after sowing in 2021 and 38, 47, 57, 81, and 118 days after sowing in 2022. At the Linying site, they were measured 18, 25, 44, 59, and 85 days after sowing in 2021 and 20, 32, 44, 57, and 87 days after sowing in 2022. Crop plant height was determined using a steel tape measure from the top of the new leaf to the bottom of the first node for maize, and from the top of the new leaf to the hypocotyl for soybean. Leaf area was captured using a mobile phone and analyzed using ImageJ software (Cosmulescu et al., 2020; Martin et al., 2020). The plant samples were decomposed into different organs, oven-dried at 105°C for 30 min to destroy the tissues, and then dried at 80°C until the weight was constant before weighing.

#### 2.3.2 Crop yield

When maize and soybean reached maturity, they were harvested to measure the grain yield and yield components. Twenty maize plants (10 plants per row) and 15 soybean plants were selected consecutively in a complete strip from each plot to determine the grain number per plant and 100-grain weight. Maize and soybean grains were sun-dried until they reached a water content of 12%.

#### 2.3.3 Photosynthetically active radiation

**Figure 2:**
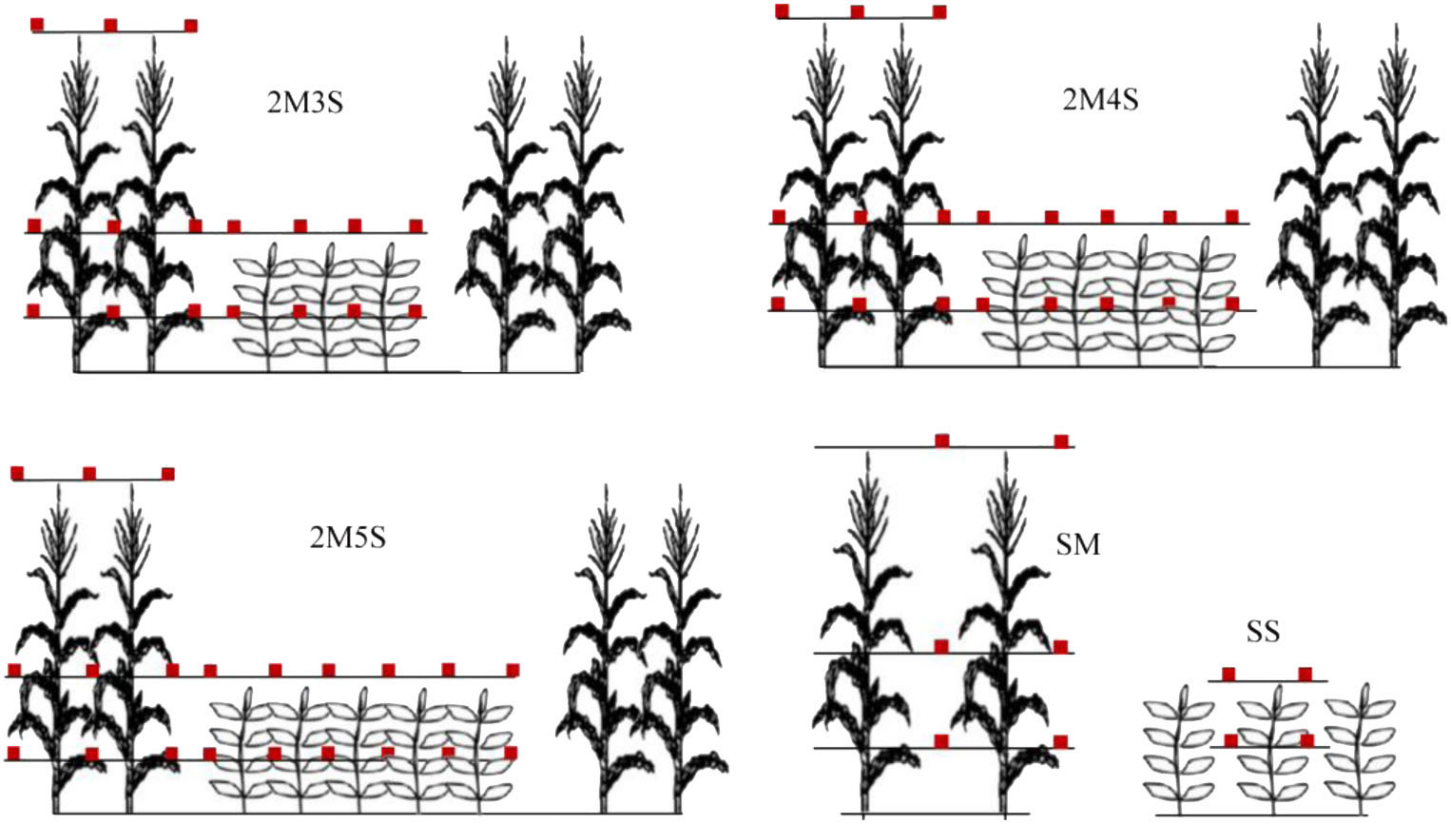
Measurement positions of light intensity (HOBO data loggers) in the intercropped and sole treatments.

HOBO UA-002-08 data loggers (Onset Computer Corporation, Bourne, MA, USA) were used to continuously monitor the light intensity of maize and soybean canopies (Hanming et al., 2012; Zhang et al., 2016). The field installation positions of the HOBO data loggers are shown in **Figure 2**. Three layers of maize canopy and two layers of soybean canopy were installed in the vertical direction. In the horizontal direction, each row of crops was also installed. At the Baotou site, the monitoring time of the HOBO data was automatically recorded every 15 min from July 30 to September 30, 2021, and from July 27 to September 30, 2022. At the Linying site, the monitoring time of the HOBO data was automatically recorded every 15 min from July 26 to September 30, 2021, and from August 1 to September 20, 2022.

We further converted the HOBO data (measured in lux) to photosynthetically active radiation (measured in µmol·m^-2^·s^-2^), with a conversion coefficient of 0.0185. The relationship between the photosynthetic active radiation and light intensity measured by the HOBO data loggers is shown in Equation 1.

### 2.4 Model construction and data analysis

#### 2.4.1 Model description

The light interception of maize and soybean was quantified using multiple regression models. The fraction of light interception for maize and soybean was calculated using Simpson’s numerical integration method (Chen et al., 2021). The specific modeling process is as follows:

#### 2.4.2 Quantifying crop light interception

The solar elevation angle (h) accurately reflects the differences in time and light intensity among regions. There was a significant heterogeneity in light over time within the intercropping system. In the first step, we establish a polynomial model to determine the relationship between h and photosynthetically active radiation (PAR) and identify h as an eigenvalue of the model. In the maize light interception model, LAD plays a crucial role in its light interception. Therefore, the LAD was used as the model eigenvalue in this case. In the soybean light interception model, soybean is influenced by the upper leaves of the neighboring maize. Hence, the upper LAD of the neighboring maize, along with its own LAD, was also included as model eigenvalues.

##### 2.4.2.1 PAR data conversion

Based on the fact that the field data obtained by the HOBO were all light intensity data in Lux unit, we firstly transformed the source data into PAR (μmol·m^-2^·s^-2^).


(1) y=0.0185×x

Where *y* is PAR (μmol·m^-2^·s^-2^) and x is light intensity (lux) measured by HOBO.

##### 2.4.2.2 The polynomial fit of the sine(h) to the logarithmic value of PAR

A polynomial model of PAR versus h was constructed to establish a relationship between h and PAR. First, the daily average light intensity for each layer in the vertical direction of the maize and soybean canopies was obtained. The value of h in the model eigenvalue was then transformed into a sine value. The logarithmic value of the objective function of light intensity for the model was used, and a polynomial model of the logarithmic value of PAR versus sine (h) was constructed based on Equation 2.


(2) $$f\left(x\right)=a\times x^{2}+b\times x+c$$

where f(x) is the logarithm of the daily mean PAR of each point in the vertical direction of the maize and soybean plants, x is sine(h), and *a*, *b*, and *c* are model parameters.

##### 2.4.2.3 Growth function for fitness the crop plant height and the LAI dynamic changes

To obtain dynamic growth data of the crop height, soybean and maize plant heights were fitted using logistic Equation 3 based on Chavan (2020) (Chavan, 2020).

Logistic function:


(3) $$f\left(t\right)=\;K\times exp\left(r\times \left(t-t_{0}\right)\right)\times p_{0}/\left(\left(K+exp\left(\left(r\times \left(t-t_{0}\right)-1\right)\right)\times p_{0}\right)\right)$$

where f(t) is the plant height, *t* is the day after sowing, *t_0_* is the initial time, *P_0_* is the initial value of the plant height, *K* is the capacity, and *r* is the rate of increase.

To obtain the dynamic growth data of the crop LAI, calculated by Equation 5, soybean and maize LAI were fitted using the beta function (4) (Yin et al., 2003).

Beta function:


(4) $$f\left(x\right)=a\times (1+\left(t_{e}-x\right)/\left(t_{e}-t_{m}\right))\times {\left(x/t_{e}\right)}^{\frac{t_{e}}{t_{e}-t_{m}}}$$

Where f(x) is the crop LAI, x is the day after sowing, *a* is the maximum LAI, *t_m_* is the time at which the maximum growth rate is reached, and *t_e_* is the time at the end of the growth period.


(5) $$LAI=L_{perplant}\times Density_{crop}/S_{area}$$

Where LAI is the crop leaf area index, $L_{perplant}\;$ is the leaf area per plant, *Density_crop_* is the crop planting density, and *S_area_* is crop area of land occupied.

##### 2.4.2.4 Maize light interception model

To obtain the distribution of light interception in the vertical direction of maize, a multivariate nonlinear model (Equation 6) was constructed using the logarithm of PAR, sin(h), and the maize LAD, calculated according to Equation 7. During model construction, 80% of the data were used to train the model, and the remaining 20% were used to validate the model.


(6) $$f\left(x\right)=\beta_{0}+\beta_{1}x_{1}+\beta_{2}x_{2}+\beta_{3}x_{1}^{2}+\beta_{4}x_{1}x_{2}+\beta_{5}x_{2}^{2}+\epsilon$$

*Wh*ere f(x) is the logarithm of PAR; *x_1_* is the sine (h); *x_2_* is the maize LAD; *β_1_*, *β_2_*, *β_3_*, *β_4_*, and *β_5_* are the model parameters, and *ϵ* is the model error.


(7) $$LAD=LAI\times H_{i}$$

Where *LAD* is the crop leaf area density and $H_{i}$ is the crop relative height.

##### 2.4.2.5 Soybean light interception model

To obtain the distribution of light interception in the vertical direction of soybean, a multivariate linear model (Equation 8) was constructed using the logarithm of PAR, sine(h), and the upper maize and soybean LADs calculated according to Equation 7. During model construction, 80% of the data were used to train the model, and the remaining 20% was used to validate the model.


(8) $$f\left(x\right)=\beta_{0}+\beta_{1}x_{1}+\beta_{2}x_{2}+\beta_{3}x_{3}+\epsilon$$

Where f(x) is the logarithm of PAR; *x_1_* is the sine(h); *x_2_* is the upper maize LAD; *x_3_* is the soybean LAD; *β_1_*, *β_2_*, and *β_3_* are the model parameters; and *ϵ* is the model error.

#### 2.4.3 Calculation the fraction of crop light interception

By completing the aforementioned processes described in Section 2.4.1, we were able to determine the distribution of light interception in the vertical direction of maize and soybean plants. We calculated the relative PAR at two end positions (a, b) and the middle point ((a+b)/2) of the crop as per Equation 9 and then determined the fraction of maize and soybean light interception via Simpson’s numerical integration method, as described in Equation 10.

##### 2.4.3.1 Relative PAR


(9) $$PAR_{R}=(PAR_{u}-PAR_{l})/PAR_{u}$$

Where $PAR_{u}$ is the PAR at the top of crop and $PAR_{l}$ is the PAR at the bottom of crop.

##### 2.4.3.1 Simpson integral


(10) $$\int_{a}^{b}f\left(x\right)dx=\left(b-a\right)/6\times \left[f\left(a\right)+4f\left(\left(a+b\right)/2\right)+f\left(b\right)\right]$$

Where *a* is the position at the bottom of the crop plant, *b* is the position at the top of the crop plant, *(a+b)/2* is the position at the middle of the crop plant, *f(a)* is the relative PAR at the bottom of the crop plant, *f(b)* is the relative PAR at the top of the crop plant, and *f((a+b)/2)* is the relative PAR at the middle of the crop plant.

#### 2.4.4 Model evaluation


(11) $$R^{2}=1-{\sum_{i=1}^{m}\left(\hat{Y_{i}}-Y_{i}\right)}^{2}/\sum_{i=1}^{m}{\left(\bar{Y}-Y_{i}\right)}^{2}$$

(12) $$MAE=1/m\sum_{i=1}^{m}\left|Y_{i}-\hat{Y_{i}}\right|$$

(13) $$RMSE=\sqrt{1/m\sum_{i=1}^{m}{\left(\hat{Y_{i}}-Y_{i}\right)}^{2}}$$

where $\;\hat{Y_{i}}$ , $\;Y_{i}$ , and $\bar{Y}$ are the simulated, observed and the mean of the observed values, respectively, and *m* is the number of data samples. If the Mean Absolute Error (MAE) and Root Mean Squared Error (RMSE) values are lower, and the R-squared (R^2^) value is higher, the model performs better.

#### 2.4.5 Data analysis

Preprocessing of the light intensity data was completed using Microsoft Excel 2019. The data of solar elevation angle was download from the web of https://pvpmc.sandia.gov/. The Logistic and Beta models to fit maize and soybean growth and multiple regression light interception models were completed using Python (3.10) platform. One-way ANOVA was performed on the relevant data using the package ‘agricolae’ in R 4.2.2.

## 3 Results

### 3.1 Crop plant height

**Figure 3:**
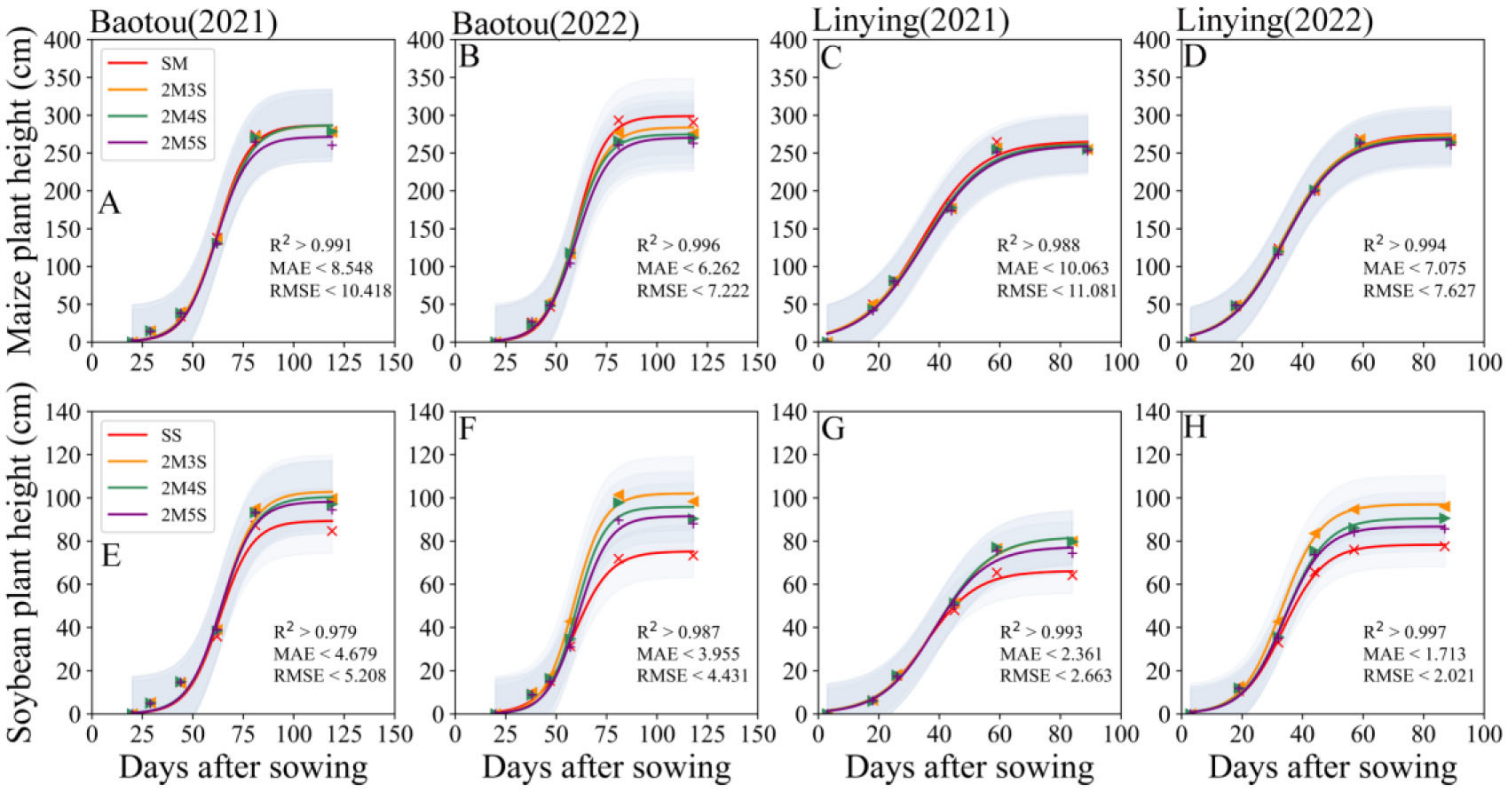
Dynamic changes in maize and soybean plant heights in different field configurations from sowing to harvest. **(A, B, E, F)** represent the Baotou site in 2021 and 2022, and **(C, D, G, H)** represent the Linying site in 2021 and 2022. The confidence intervals are all one time of the SD.

As shown in **Figure 3**, the plant heights of maize and soybean were fitted with high accuracy, with model R^2^ greater than 0.979, MAE< 10.063, and RMSE< 11.081 (**Figure 3**). The plant height of the intercropped maize and soybean decreased with increasing soybean row number. Compared with 2M3S, the maize plant height in the 2M5S treatment decreased by 5.19% and 4.82% in 2021 and 2022 at the Baotou site (**Figures 3A, B**), and 1.30% and 2.05% in 2021 and 2022 at the Linying site, respectively (**Figures 3C, D**). Furthermore, compared with 2M3S, the plant height of soybean in the 2M5S treatment decreased by 4.40% and 10.23% in 2021 and 2022, respectively, at the Baotou site (**Figures 3E, F**), and 5.70% and 10.57% in 2021 and 2022, respectively at the Linying site (**Figures 3G, H**).

### 3.2 Crop LAI

**Figure 4:**
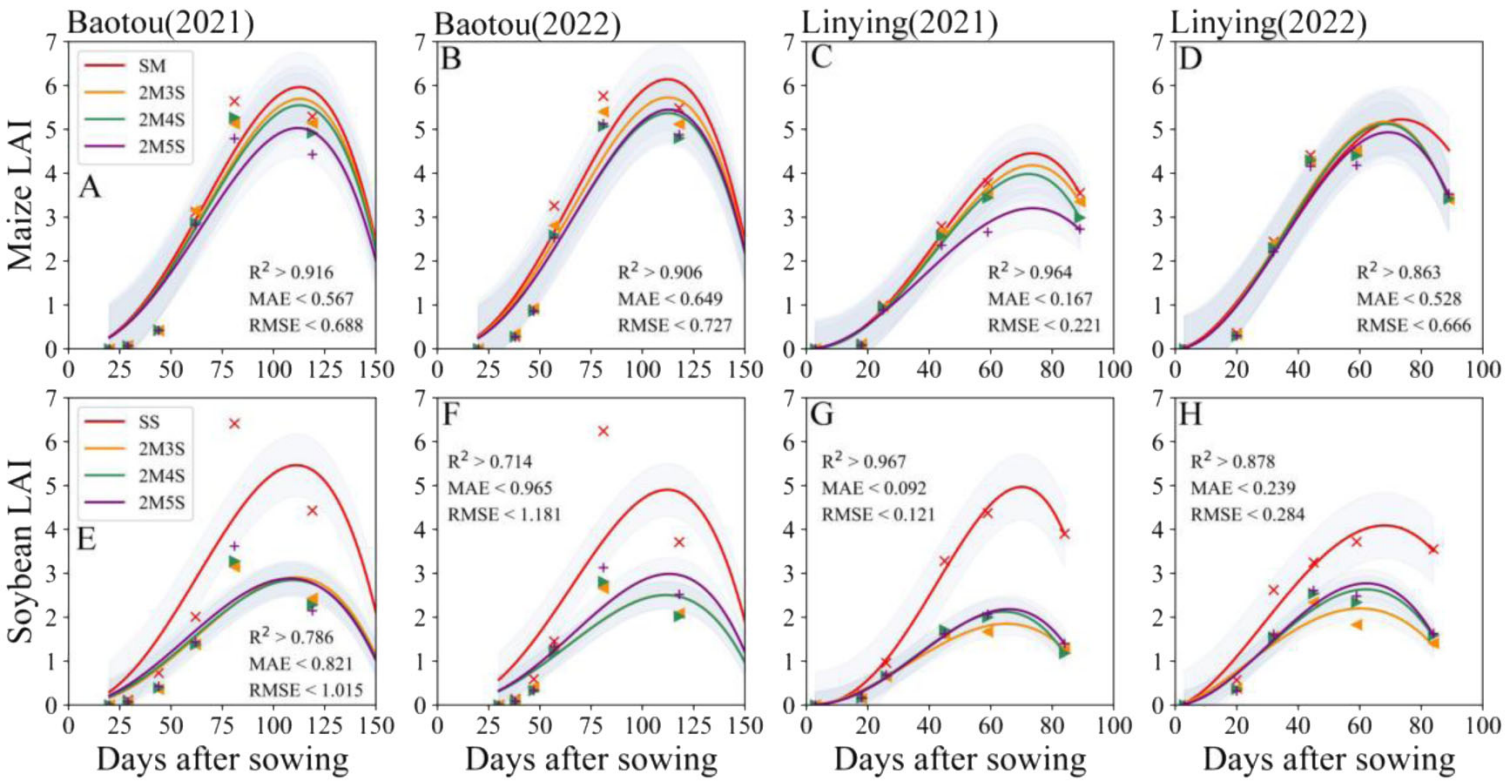
Dynamic changes in maize and soybean LAI in different field configurations from sowing to harvest. **(A, B, E, F)** represent the Baotou site in 2021 and 2022, and **(C, D, G, H)** represent the Linying site in 2021 and 2022. The confidence intervals are all one time of the SD.

The LAI of maize and soybean was better fitted, with R^2^ > 0.714, MAE< 0.965, and RMSE< 1.181 (**Figure 4**). The LAI of intercropped maize decreased with increasing soybean row numbers. Compared to 2M3S, the LAI of maize in the 2M5S treatment decreased by 11.60% and 4.89% in 2021 and 2022 at the Baotou site (**Figures 4A, B**), and 23.44% and 5.57% in 2021 and 2022 at the Linying site, respectively (**Figures 4C, D**). In contrast, the LAI of intercropped soybean increased with increasing soybean row numbers. Compared to 2M3S, the LAI of soybean in the 2M5S treatment increased by 19.12% in 2022 at the Baotou site (**Figure 4F**), and 17.84% and 24.77% in 2021 and 2022 at the Linying site, respectively (**Figures 4G, H**).

### 3.3 Construction of the crop light interception model

#### 3.3.1 Maize light interception model

**Figure 5:**
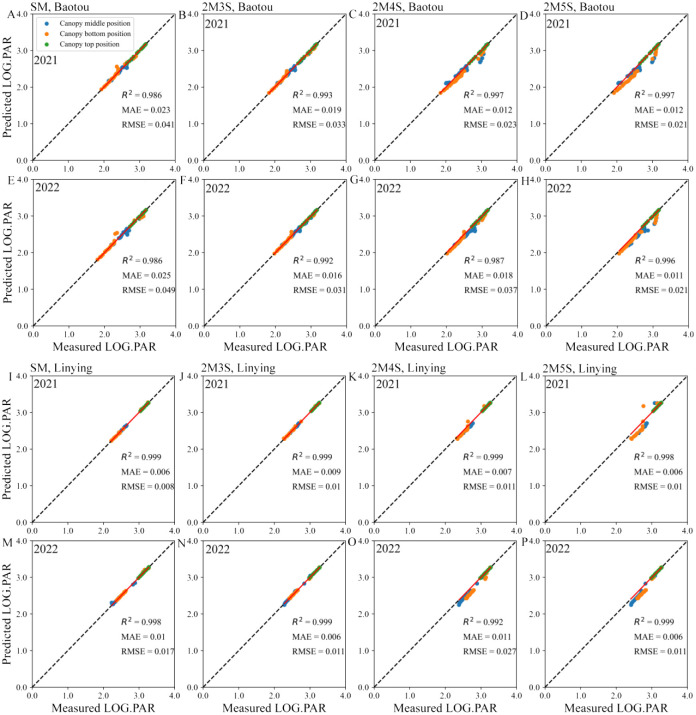
Verification of maize light interception models for different field configurations. **(A–H)** represent the Baotou site in 2021 and 2022, and **(I–P)** represent the Linying site in 2021 and 2022. LOG PAR represents logarithm value of PAR.

As shown in **Supplementary Table S1**, there was a good polynomial fit between the logarithm of PAR and the sine of the solar angle (h), with an R² greater than 0.70. Based on this, we calculated the predicted PAR at different positions within the maize canopy, and found a positive correlation between the measured PAR and the predicted PAR, with R^2^ > 0.986, MAE< 0.025, and RMSE< 0.049 (**Figure 5**). This indicated that the model was reliable.

#### 3.3.2 Soybean light interception model

**Figure 6:**
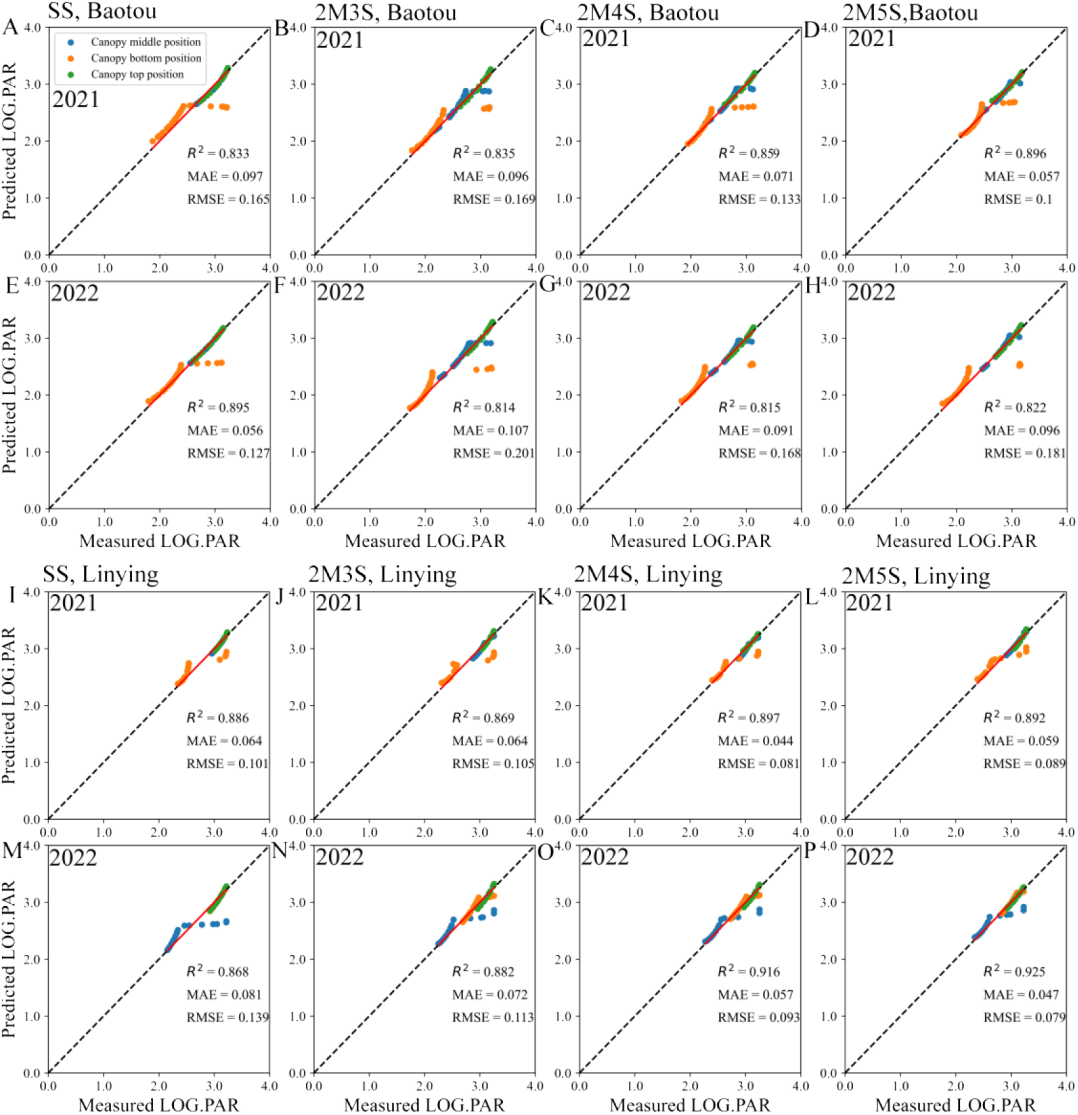
Verification of soybean light interception models for different field configurations. **(A–H)** represents the Baotou site in 2021 and 2022, and **(I–P)** represents the Linying site in 2021 and 2022. LOG PAR represents logarithm value of PAR.

As shown in **Supplementary Table S2**, there was also good polynomial fit between the logarithm of PAR and sine(h), with R^2^ > 0.62. Based on this, we calculated the predicted PAR at different positions within the soybean canopy and found that the measured PAR and the predicted PAR were positively correlated, with R^2^ > 0.814, MAE< 0.107, and RMSE< 0.201 (**Figure 6**). This indicated that the model was reliable.

### 3.4 Fraction and cumulative light interception of crop

**Figure 7:**
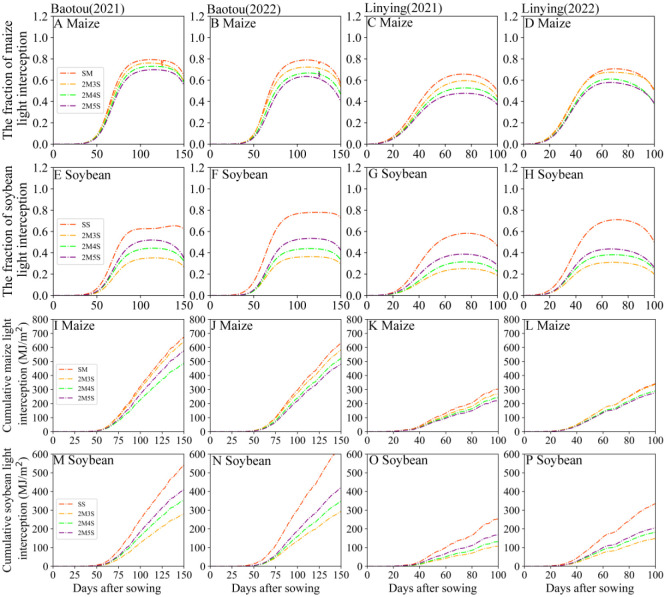
Fraction of light interception and accumulative light interception of maize and soybean for different field configurations from sowing to harvest. **(A–D)** represent the fraction of maize light interception at the Baotou and Linying sites in 2021 and 2022, respectively. **(E–H)** represent the fraction of soybean light interception at the Baotou and Linying sites in 2021 and 2022, respectively. **(I–L)** represent the cumulative light interception of maize at the Baotou and Linying site in 2021 and 2022. **(M–P)** represent the cumulative light interception of soybean at the Baotou and Linying sites in 2021 and 2022, respectively.

As shown in **Figure 7**, compared to monoculture, the fraction of light interception in intercropped maize and soybean decreased, and the mean fraction of light interception in intercropped maize was lower 11.42% in Baotou and 16.82% in Linying compared to sole maize, and 41.60% lower in Baotou and 46.79% lower in Linying than in sole soybean throughout both years from seeding to harvest. Among strip intercropping treatments, the fraction of light interception in maize decreased with increasing soybean row number (**Figures 7A–D**). Compared to 2M3S, the cumulative light interception of maize in the 2M5S treatment decreased by 9.90% and 17.64% in 2021 and 2022 at the Baotou site, respectively, and 17.83% and 17.64% in 2021 and 2022 at the Linying site, respectively (**Figures 7I–L**).

In contrast, the fraction of light interception in intercropped soybean increased with increasing soybean row number. Compared to 2M3S, the fraction of soybean light interception in the 2M5S treatment increased by 50.00% and 45.45% in 2021 and 2022 at the Baotou site, respectively, and 57.14% and 36.84% in 2021 and 2022 at the Linying site, respectively (**Figures 7E–H**). The cumulative light interception of soybean in the 2M5S treatment increased by 45.75% and 43.70% in 2021 and 2022, respectively, at the Baotou site, and 55.96% and 38.39% in 2021 and 2022, respectively, at the Linying site (**Figures 7M–P**).

**Figure 8:**
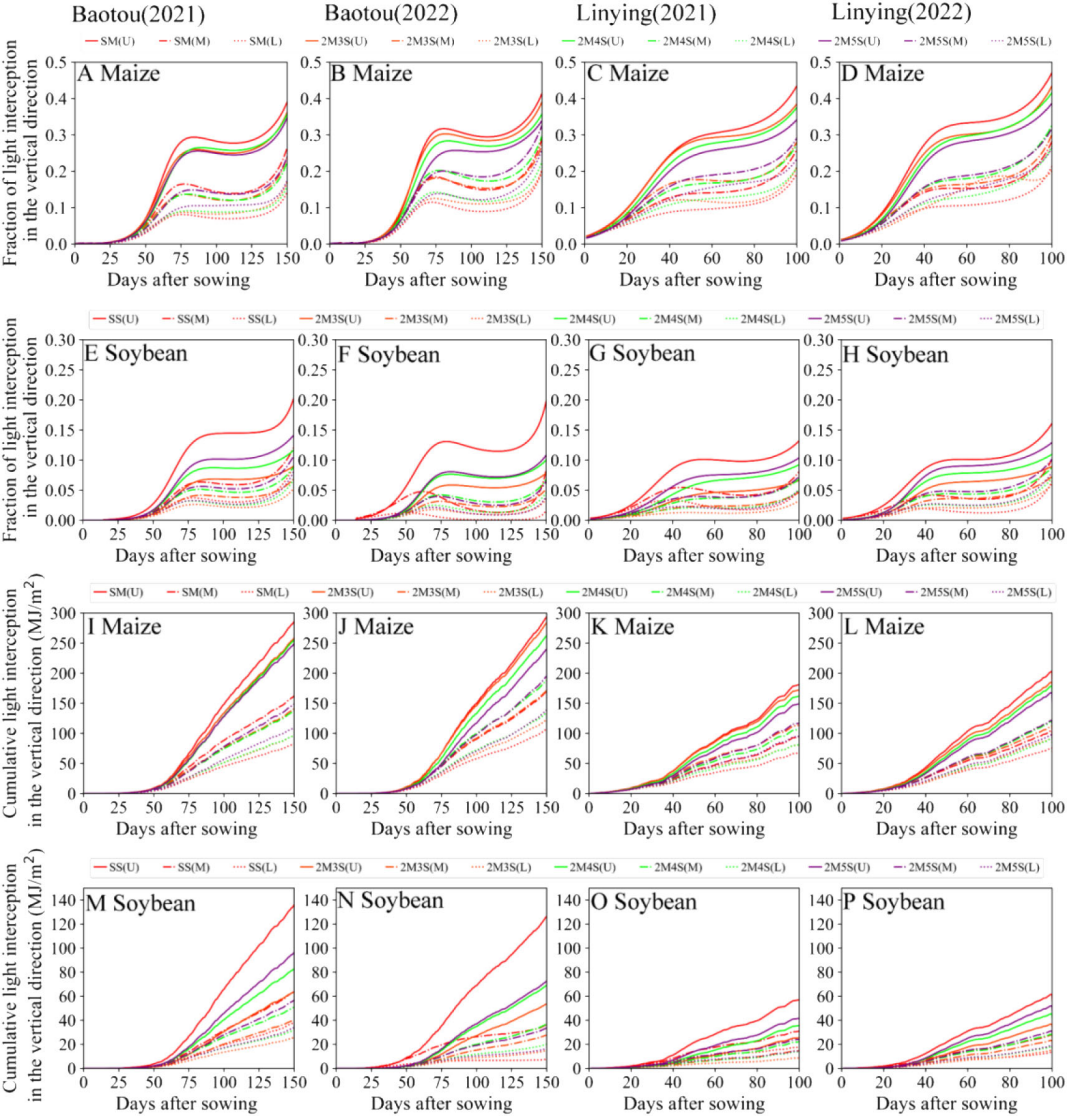
Cumulative light interception in the vertical direction of maize and soybean for different field configurations from sowing to harvest. **(A–D)** represent the fraction of light interception in the vertical direction of maize at the Baotou and Linying sites in 2021 and 2022, respectively. **(E–H)** represent the fraction of light interception in the vertical direction of soybean at the Baotou and Linying sites in 2021 and 2022. **(I–L)** represent the cumulative light interception in the vertical direction of maize at the Baotou and Linying site in 2021 and 2022, respectively. **(M–P)** represent the cumulative light interception in the vertical direction of soybean at the Baotou and Linying sites in 2021 and 2022, respectively. The crop is divided into four layers from top to bottom, where U (part one and two) represents the upper layer of the crop, M (part two and three) represents the middle layer of the crop, and L (part three and four) represents the lower layer of the crop.

As shown in **Figure 8**, compared to intercropped maize, the cumulative light interception of the lower layer in sole maize decreased by 20.17% and 21.46% in 2021 and 2022 at the Baotou site, respectively and 27.05% and 16.91% in 2021 and 2022 at the Linying site, respectively (**Figure 8I–L**). In the intercropping system, as the number of soybean rows increased, the fraction of light interception in the upper maize layer decreased, while the fraction of light interception in lower maize layer increased. Compared to 2M3S, the cumulative light interception of the upper maize layer in 2M5S decreased by 14.36% and 18.41% in 2021 and 2022 at the Baotou site, respectively and 15.38% and 11.41% in 2021 and 2022 at the Linying site, respectively. On the other hand, the cumulative light interception of the lower maize layer in 2M5S increased by 10.34% and 8.15% in 2021 and 2022, respectively at the Baotou site, and 9.02% and 7.06% in 2021 and 2022, respectively, at the Linying site (**Figures 8I–L**).

The light interception rates of all the soybean layers increased as the number of soybean rows increased. Compared to 2M3S, the cumulative light interception of the upper soybeans layer in the 2M5S treatment increased by 50.64% and 35.81% in 2021 and 2022 at the Baotou site, respectively and 66.50% and 40.89% in 2021 and 2022 at the Linying site, respectively (**Figures 8M–P**).

### 3.5 Dry matter accumulation

**Figure 9:**
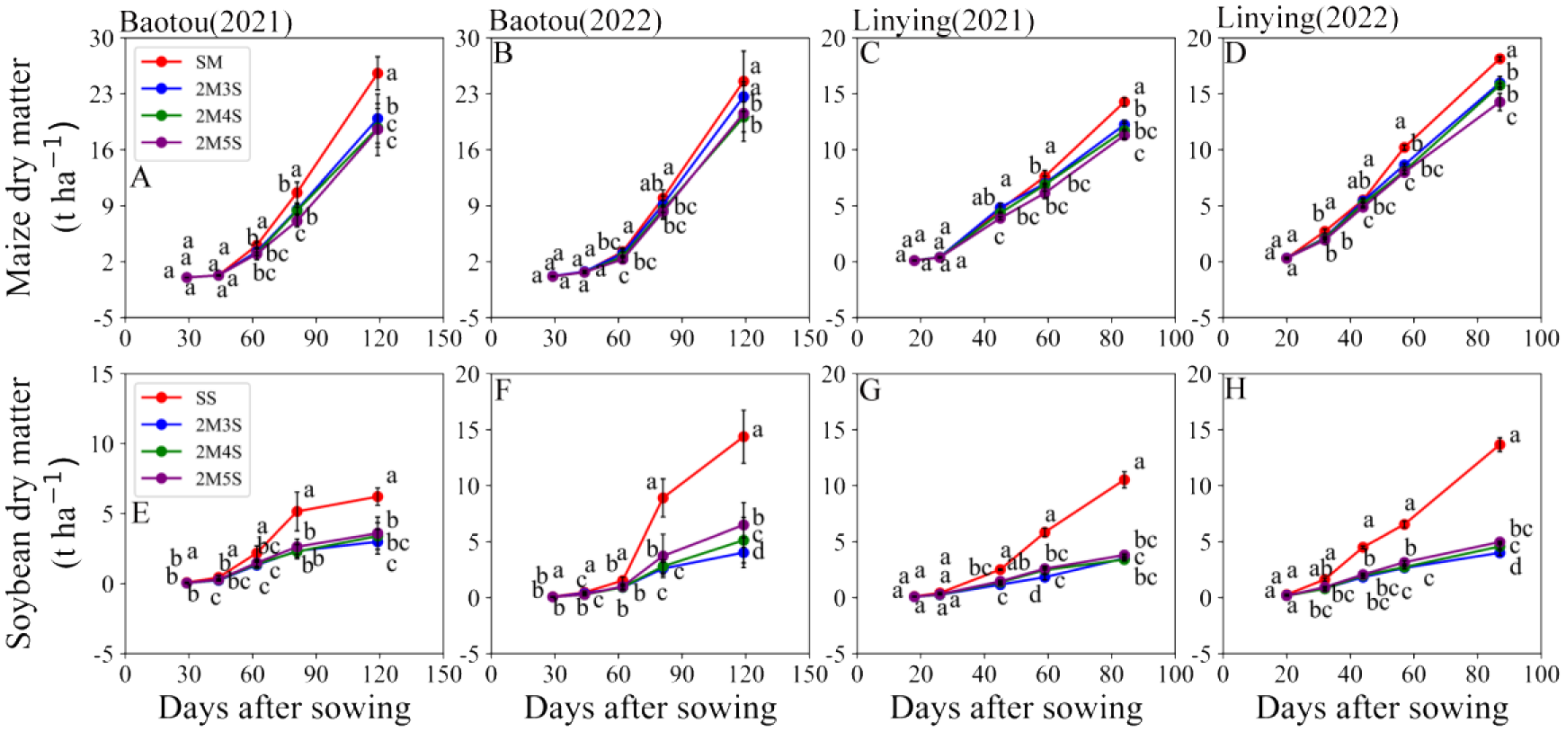
Dry matter accumulation in maize and soybean in different field configurations. **(A, B, E, F)** represent the Baotou site in 2021 and 2022. **(C, D, G, H)** represent the Linying site in 2021 and 2022.

As shown in **Figure 9**, the dry matter accumulation of the intercropped maize was significantly lower than that of the sole maize. It showed a decreasing trend with increasing soybean row number for different measurement periods (**Figures 9A–D**). Compared to 2M3S, the dry matter of maize in the 2M5S treatment was significantly decreased by 6.86% in 2021, 119 days after sowing and 9.20% in 2022, 118 days after sowing at the Baotou site, and 7.77%in 2021, 85 days after sowing and 10.73% in 2022, 87 days after sowing at the Linying site (**Figures 9A–D**).

In contrast, the dry matter accumulation of intercropped soybean increased with increasing soybean row number for the different measurement periods (**Figures 9E–H**). Compared to 2M3S, the dry matter of soybean in the 2M5S treatment was significantly increased by 16.75%, in 2021, 119 days after sowing and 37.81% in 2022, 118 days after sowing at the Baotou site, and 21.15%, in 2021, 85 days after sowing and 19.62% in 2022, 87 days after sowing at the Linying site (**Figures 9E–H**).

### 3.6 Correlation analysis between the average fraction of crop light interception and dry matter accumulation

**Figure 10:**
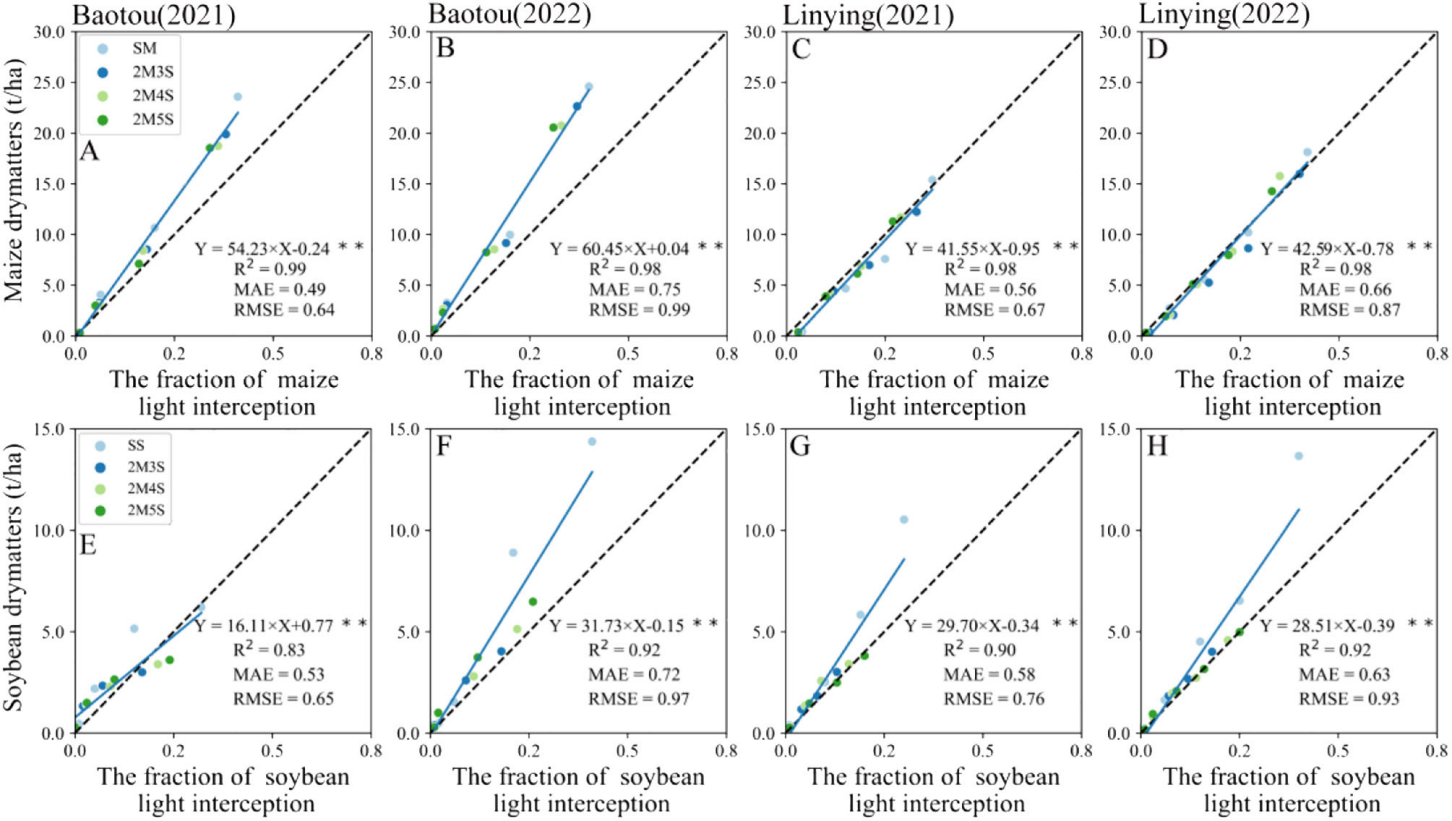
Relationship between crop dry matter and average light interception rates in different field configurations. **(A, B, E, F)** represent the Baotou site in 2021 and 2022. **(C, D, G, H)** represent the Linying site in 2021 and 2022.

As shown in **Figure 10**, there was a significant linear relationship between maize and soybean dry matter accumulation and annual average light interception. In both sites and for both years (2021 and 2022), the model R^2^ for maize were higher than 0.98 (**Figures 10A–D**), and the model R^2^ for soybean were more than 0.83 (**Figures 10E–H**).

### 3.7 Yield

As shown in **Table 2**, the yields of intercropped maize and soybean were significantly lower than those of corresponding monocrops. In the strip intercropping system, maize yield decreased with increasing soybean row number. Compared to 2M3S, the maize yield in the 2M5S treatment was significantly decreased by 8.06% and 4.00% in 2021 and 2022 at the Baotou site, and 5.15% and 7.17% in 2021 and 2022, respectively, at the Linying site. In contrast, soybean yield showed an increasing trend with increasing number of soybean row number. Compared to 2M3S, soybean yield in 2M5S increased by 27.86% and 19.51% in 2021 and 2022 at Baotou site, and 29.10% and 27.93% in 2021 and 2022, respectively, at the Linying site.

**Table 2:** Maize, soybean, and system yields affected by configuration in maize soybean strip intercropping.

Site	Treatments	2021			2022		
		Maize yield (t ha^-1^)	Soybean yield (t ha^-1^)	System yield (t ha^-1^)	Maize yield (t ha^-1^)	Soybean yield (t ha^-1^)	System yield (t ha^-1^)
Baotou	SS	–	4.00 ± 0.03a	4.00 ± 0.03c	–	4.52 ± 0.01a	4.52 ± 0.01c
	SM	16.93 ± 0.47a	–	16.93 ± 0.47a	15.21 ± 0.41a	–	15.21 ± 0.41b
	2M3S	15.66 ± 0.30b	1.40 ± 0.01d	17.05 ± 0.30a	14.05 ± 0.12b	1.64 ± 0.03d	15.68 ± 0.16ab
	2M4S	15.24 ± 0.18c	1.66 ± 0.02c	16.90 ± 0.19a	14.04 ± 0.49b	1.83 ± 0.04c	15.87 ± 0.47a
	2M5S	14.39 ± 0.11d	1.79 ± 0.04b	16.18 ± 0.15b	13.49 ± 0.31c	1.96 ± 0.03b	15.44 ± 0.29ab
Linying	SS	–	3.31 ± 0.10a	3.31 ± 0.10c	–	3.42 ± 0.19a	3.42 ± 0.19c
	SM	8.08 ± 0.06a	–	8.08 ± 0.06b	8.88 ± 0.07a	–	8.88 ± 0.07b
	2M3S	7.68 ± 0.06b	1.34 ± 0.04d	9.02 ± 0.06a	8.24 ± 0.08b	1.11 ± 0.01c	9.35 ± 0.06a
	2M4S	7.44 ± 0.05c	1.70 ± 0.02bc	9.14 ± 0.08a	8.06 ± 0.06b	1.37 ± 0.06bc	9.43 ± 0.11a
	2M5S	7.28 ± 0.12c	1.73 ± 0.01b	9.01 ± 0.12a	7.65 ± 0.16c	1.42 ± 0.03b	9.07 ± 0.17b

Data are expressed as the mean of three replicates ± standard error (n = 3). Values followed by different letters within a column are significantly different (P< 0.05).

## 4 Discussion

### 4.1 Effect of intercropping configuration on crops

Crop phenotypes are influenced by environmental factors (Gratani, 2014). The Northwest and Yellow-Huai-Hai regions are the main cultivation areas for maize-soybean strip intercropping. However, owing to different climatic conditions, the optimal configuration of maize-soybean strip intercropping is still unclear. Compared to sole cropping, intercropped crops exhibit a competitive advantage due to significant spatiotemporal differences (Gebru, 2015). In cereal-based intercropping systems, two rows of maize obtain maximum light interception due to the border row effect (Wang et al., 2017, 2021b). The row configuration in this study also confirmed this conclusion: taller crops have a competitive advantage over lower ones in terms of light resource competition. In a maize-soybean strip intercropping system, Liu et al. (2017b) suggested that reducing the distance between two rows of maize to 20 cm led to a decrease in the height and LAI of intercropped maize. Ren et al. (2016) observed a decrease in the height and LAI of maize when reducing the maize planting proportion. The research findings mentioned above indicate that reducing the row spacing or planting proportion of maize decreases its growth space, intensifies intra-specific competition, and affects the plant height and LAI (Lithourgidis et al., 2011; Liang et al., 2023). In the present study, as the soybean rows increased, the growth space of maize decreased, resulting in a decline in plant height and LAI. This is consistent with the results of previous studies (Liu et al., 2017b). However, as the number of soybean rows increased, the annual average reduction in the maize plant height and LAI at the Baotou site was 5.01% and 8.24%, respectively, with reduction magnitudes 2.99 and 0.57 times higher than that observed in maize at the Linying site. This can be attributed to intensified intra-specific competition due to increased density, as well as the influence of maize variety. Maize height and LAI are important factors in calculating light interception; therefore, as soybean rows increased, the light interception of maize showed a decreasing trend. These findings are consistent with those reported by Liu et al. (2017b) and Wu et al. (2021). However, the maize plant height and LAI at the Baotou site were 1.06 and 1.26 times higher than those at the Linying site, respectively. This resulted in an overall increase in the light interception efficiency of intercropped maize at the Baotou site compared to that at the Linying site. The spatial light interception results of maize further indicate that the decrease in the light interception rate of intercropped maize compared to the sole is primarily caused by the reduced light interception of the upper-level maize plants. Although the light interception rate of the lower-level maize plants increased, it could not compensate for the light loss of the upper-level maize plants.

Xue et al. (2015) found a linear relationship (R^2^ = 0.98) between light interception and cotton biomass. There is a large difference in climate resources between the two sites, and there is also a large difference in conditions such as water and fertilizer needed to satisfy crop growth. To eliminate the adverse effects of water and fertilizer on the experimental results, we applied fertilizer according to the target maize yield. In this study, there was a highly significant linear relationship (R^2^ > 0.98) between intercropped maize’s biomass accumulation and the light interception rate. Appropriate soybean rows facilitate coordinated competition to increase dry matter accumulation and yield in intercropping systems (Mahallati et al., 2015; Raza et al., 2020; Van Oort et al., 2020). In a maize-soybean intercropping system with a 2-meter soybean rows, reducing the row spacing of maize from 60 to 20 cm significantly decreased biomass accumulation by 12.09% (Liu et al., 2017b). In this study, maize biomass accumulation showed a significant decreasing trend as the number of soybean rows increased, but the magnitude or reduction was relatively small. This could be attributed to the suitability of the larger soybean rows used in this study for maize production. The final yield followed the same pattern as biomass accumulation. When the number of soybean rows increased from three rows to five rows, the annual average maize yield decreased by 6.03% at the Baotou site and 6.16% at the Linying site, the decrease in maize yield by 0.13% at the Baotou site compared to the Linying site is primarily associated with the compensatory effect of maize density.

For the low crop soybean, the shade response to prolonged shade by maize significantly increased plant height and significantly reduced dry matter accumulation and yield compared to monoculture (Wu et al., 2017). In this study, intercropped soybean exhibited an increase in plant height and a decrease in LAI due to shading. Fan et al. (2018) found that in a traditional soybean-maize intercropping system (1:1) with a 1-meter soybean rows, soybean had no significant difference in plant height compared to sole cropping in the later stages of growth due to severe shading. However, in a strip intercropping system with a 2-meter soybean rows, where the growing space for intercropped soybean was larger and shading was reduced, soybean showed a significant increase in plant height compared to sole cropping. In our study, the soybean rows used was greater than 2.2 meters, resulting in relatively light shading on intercropped soybean, which maintained higher plant height compared to sole cropping but decreased as the soybean rows increased. As the number of soybean rows increased, the annual average reduction in soybean plant height and LAI at the Baotou site was 7.32% and 6.17%, respectively, with reduction magnitudes 1.05 and 0.29 times higher than that observed in soybean at the Linying site. On the one hand, this is a result of maize shading, while on the other hand, it is related to differences in soybean varieties and meteorological conditions.

The light interception of intercropped soybean is closely related to the LAD of the neighboring maize and its own growing space (Feng et al., 2019). In our study, the LAD of neighboring maize and soybean was an important factor in calculating the light interception of strip intercropped soybean. Increasing the soybean rows favored higher light interception by soybean, and the increase in light interception rate of intercropped soybean at the Baotou site compared to the Linying site was attributed to the respective increases in the soybean plant height and LAI by 1.16 and 1.21 times. The spatial light interception results for soybean further indicate that upper-level intercropped soybean contributes the most to the overall soybean canopy. When the number of soybean rows increased from three to five, the annual average soybean yield increased by 23.69% at the Baotou site and by 28.52% at the Linying site. This finding is consistent with the results of the study by Wang et al. (2021b), where the 4.83% increase in soybean yield at the Linying site compared to the Baotou site was primarily attributed to the reduced shading effect from lower maize density on soybean. Previous research indicated that in a traditional soybean-maize intercropping system with a 1-meter soybean rows, soybean biomass and yield significantly decreased by 59.71% and 54.33%, respectively, owing to severe shading. However, in a strip intercropping system with a 2-meter soybean rows, soybean biomass and yield significantly increased as the growing space for soybean increased (Liu et al., 2018). This is consistent with the changes observed in soybean biomass and yield in our study.

### 4.2 Quantification of intercropping light interception

Given that previous studies on light interception in intercropping systems have focused on the diurnal variation of instantaneous light interception, these findings may not accurately reflect the temporal and spatial variations in light interception in such heterogeneous canopies. This study developed a new method to quantify light interception in strip intercropping, addressing the lack of quantitative approaches to crop light interception in such systems. The newly developed quantitative model for light interception in soybean-maize intercropping demonstrated R^2^ values exceeding 0.814, MAE< 0.107, and RMSE< 0.201. The accuracy of this model surpassed that of the study by Munz et al. (2014), which simulated light interception in bush bean (*Phaseolus vulgaris L.* var. *nana*) using the geometric model of the strip-intercropping system developed by (Gijzen and Goudriaan, 1989). To ensure high accuracy, the model introduces key parameters h and crop LAD. The parameter h not only better reflects the parameter extinction coefficient (*K*) needed for the model (Campbell, 1990; Campbell and Norman, 2000), but also accurately reflects the different spatial and temporal differences (Ezeilo, 1979).

Crop varieties alter canopy light distribution (Niinemets, 2010), and only one maize and soybean variety was used for modeling in this study, which seems to contradict the requirement of diversifying data sources for modeling. In fact, crop varieties influence canopy light distribution mainly through the LAI and leaf angle size (Li et al., 2021b). Previous two-dimensional light interception models did not consider crop varieties but quantified intercrop light interception by indirectly considering LAI parameters (Zhang et al., 2008; Wang et al., 2015). In this study, intercrop LAD and h were used as important parameters of the model based on previous studies, which will improve the problem that the previous light interception models could not describe the light interception by crop varieties. Thus, the model quantifies light interception in strip intercropping under large-scale conditions with knowledge of the crop LAD.

Previous studies evaluating models for light interception in heterogeneous environments were predominantly based on Beer’s law (Sinoquet and Bonhomme, 1991; 1992; Pronk et al., 2003) and subsequently revised and developed further (Zhang et al., 2008; Wang et al., 2015). These models have been applied to calculate light interception in soybean-maize strip intercropping (Liu et al., 2017b). However, these models neglect the internal light transmission within the crop canopy when calculating light interception in strip intercropping. Moreover, it is difficult for these models to quantify the diurnal variations in light interception in strip intercropping (Wang et al., 2015). Therefore, the method developed in this study effectively addresses the limitations of previous research on quantifying light interception in strip intercropping. However, there is room for improvement in this model. To enhance modeling efficiency, the next step involves integrating spatiotemporal light intensity data from different sources and optimizing model parameters to achieve the goal of quantifying crop light interception in strip intercropping.

## 5 Conclusion

Row configuration significantly affected the growth, dry matter, and yield of intercropped maize and soybean. Increasing the number of soybean rows led to an increase in light interception of the lower layer of maize, but it could not compensate for the loss of light interception by the upper layer of maize. The newly created model exhibited high accuracy in predicting the variations of spatial-temporal light interception, which was further verified by observing changes in dry matter and yield across different configurations. The highest system yield was observed for 2M4S, indicating that the pursuit of system benefits would become the target for optimizing configurations in strip intercropping.

## Data Availability

The original contributions presented in the study are included in the article/**Supplementary Material**. Further inquiries can be directed to the corresponding author/s.

## Supplementary material

The attached tables show the relationship between the LOG values of PAR and the sine of the solar altitude angle for the upper, middle, and lower layers of maize and soybean, respectively.
