# Supplementary material for: Quantification of spatial-temporal light interception of crops in different configurations of soybean-maize strip intercropping
Source: Front Plant Sci. 2024 Sep 30;15:1376687. doi: 10.3389/fpls.2024.1376687 (PMC11471477; doi:10.3389/fpls.2024.1376687)
Supplement: Supplementary file 1 [file Table1.docx]

| **Table 1S. Polynomial fitting of the logarithm of maize canopy light intensity and sine value of solar elevation angle** | | | | | | | | | | | | | | | | | | | | | | |
| --- | --- | --- | --- | --- | --- | --- | --- | --- | --- | --- | --- | --- | --- | --- | --- | --- | --- | --- | --- | --- | --- | --- |
| Site | Year | Treatments | The top of canopy | | | |  | The middle of canopy | | | |  | The bottom of canopy | | | | | | | | | |
|  |  |  | a | b | c | R^2^ |  | a | b | c | R^2^ |  | a | | b | | c | | R^2^ | |  |  |
| Baotou | 2021 | SM | -2.62 | 4.32 | 1.53 | 0.73 |  | -1.6 | 3.19 | 1.21 | 0.72 |  | -1.9 | | 3.46 | | 0.97 | | 0.75 | |  |  |
|  |  | 2M3S | -2.62 | 4.32 | 1.53 | 0.73 |  | -1.66 | 3.2 | 1.25 | 0.71 |  | -1.24 | | 2.86 | | 1.07 | | 0.71 | |  |  |
|  |  | 2M4S | -2.62 | 4.32 | 1.53 | 0.73 |  | -1.68 | 3.52 | 1.09 | 0.79 |  | -1.66 | | 3.6 | | 0.86 | | 0.75 | |  |  |
|  |  | 2M5S | -2.62 | 4.32 | 1.53 | 0.73 |  | -0.84 | 2.83 | 1.26 | 0.78 |  | -1.01 | | 2.97 | | 1.1 | | 0.72 | |  |  |
|  | 2022 | SM | -1.87 | 3.39 | 1.81 | 0.72 |  | -1.72 | 3.09 | 1.41 | 0.72 |  | -1.66 | | 3.16 | | 0.99 | | 0.76 | |  |  |
|  |  | 2M3S | -1.87 | 3.39 | 1.81 | 0.72 |  | -1.73 | 3.29 | 1.34 | 0.75 |  | -1.27 | | 2.8 | | 1.23 | | 0.73 | |  |  |
|  |  | 2M4S | -1.87 | 3.39 | 1.81 | 0.72 |  | -1.31 | 3.15 | 1.39 | 0.73 |  | -1.66 | | 3.13 | | 1.21 | | 0.72 | |  |  |
|  |  | 2M5S | -1.87 | 3.39 | 1.81 | 0.72 |  | -1.48 | 2.88 | 1.58 | 0.72 |  | | -1.21 | | 2.71 | | 1.33 | | 0.72 | |  |
| Linying | 2021 | SM | -2.17 | 4.03 | 1.47 | 0.73 |  | -1.54 | 3.3 | 1.16 | 0.72 |  | -2.31 | | 4.04 | | 0.78 | | 0.78 | |  |  |
|  |  | 2M3S | -2.17 | 4.03 | 1.47 | 0.73 |  | -1.81 | 3.71 | 1.06 | 0.73 |  | -2.33 | | 4.22 | | 0.73 | | 0.78 | |  |  |
|  |  | 2M4S | -2.17 | 4.03 | 1.47 | 0.73 |  | -1.57 | 3.5 | 1.11 | 0.75 |  | -1.83 | | 3.68 | | 0.93 | | 0.73 | |  |  |
|  |  | 2M5S | -2.17 | 4.03 | 1.47 | 0.73 |  | -1.9 | 3.68 | 1.23 | 0.74 |  | -1.81 | | 3.76 | | 0.98 | | 0.77 | |  |  |
|  | 2022 | SM | -2.06 | 4 | 1.44 | 0.77 |  | -1.59 | 3.37 | 1.04 | 0.74 |  | -1.86 | | 3.5 | | 0.94 | | 0.73 | |  |  |
|  |  | 2M3S | -2.06 | 4 | 1.44 | 0.77 |  | -1.7 | 3.39 | 1.11 | 0.75 |  | -1.71 | | 3.46 | | 0.97 | | 0.81 | |  |  |
|  |  | 2M4S | -2.06 | 4 | 1.44 | 0.77 |  | -1.75 | 3.37 | 1.29 | 0.73 |  | -1.61 | | 3.17 | | 1.19 | | 0.75 | |  |  |
|  |  | 2M5S | -2.06 | 4 | 1.44 | 0.77 |  | -1.24 | 2.86 | 1.43 | 0.73 |  | -1.48 | | 3.08 | | 1.22 | | 0.78 | |  |  |

Note: SS represents sole soybean with a density of 225,000 plants ha^-1^at Baotou and Linying site, SM represents single maize with a density of 75,000 plants ha^-1^ at Baotou site and 67,500 plants ha^-1^ at Linying site, 2M3S represents two rows of maize alternated with three soybean rows with a maize density of 75,000 plants ha^-1^ at Baotou site and 67,500 plants ha^-1^ at Linying site, 2M4S represents two rows of maize alternated with four soybean rows with a maize density of 75,000 plants ha^-1^ at Baotou site and 67,500 plants ha^-1^ at Linying site, 2M5S represents two rows of maize alternated with five soybean rows with a maize density of 75,000 plants ha^-1^ at Baotou site and 67,500 plants ha^-1^ at Linying site. a, b and c are model parameters, respectively, and R^2^ is the model coefficient of determination.

| **Table 2S. Polynomial fitting of the logarithm of soybean canopy light intensity and sine value of solar elevation angle** | | | | | | | | | | | | |
| --- | --- | --- | --- | --- | --- | --- | --- | --- | --- | --- | --- | --- |
| Site | Year | Treatments | The top of canopy | | | |  | The middle of canopy | | | | |
|  |  |  | a | b | c | R^2^ |  | a | b | c | R^2^ |  |
| Baotou | 2021 | SS | -3.69 | 5.25 | 1.41 | 0.73 |  | -2.62 | 4.31 | 0.79 | 0.72 |  |
|  |  | 2M3S | -2.31 | 4.04 | 1.14 | 0.78 |  | -1.16 | 3 | 0.95 | 0.72 |  |
|  |  | 2M4S | -2.39 | 4.06 | 1.25 | 0.75 |  | -1.03 | 2.41 | 1.3 | 0.62 |  |
|  |  | 2M5S | -2.47 | 4.02 | 1.43 | 0.8 |  | -2.37 | 3.7 | 1.07 | 0.66 |  |
|  | 2022 | SS | -1.97 | 3.8 | 1.58 | 0.74 |  | -2.03 | 3.87 | 0.8 | 0.75 |  |
|  |  | 2M3S | -1.61 | 3.23 | 1.43 | 0.77 |  | -1.87 | 3.1 | 0.94 | 0.75 |  |
|  |  | 2M4S | -2.21 | 3.7 | 1.44 | 0.76 |  | -1.97 | 3.26 | 1.01 | 0.66 |  |
|  |  | 2M5S | -1.7 | 3.27 | 1.6 | 0.8 |  | -2.37 | 3.79 | 0.9 | 0.75 |  |
| Linying | 2021 | SS | -2.18 | 4.01 | 1.46 | 0.71 |  | -2.44 | 4.41 | 0.84 | 0.83 |  |
|  |  | 2M3S | -3.09 | 5 | 1.11 | 0.82 |  | -2.37 | 4.23 | 0.77 | 0.73 |  |
|  |  | 2M4S | -2.65 | 4.33 | 1.34 | 0.72 |  | -2.66 | 4.41 | 0.85 | 0.73 |  |
|  |  | 2M5S | -2.15 | 4.18 | 1.32 | 0.81 |  | -2.2 | 3.96 | 0.95 | 0.73 |  |
|  | 2022 | SS | -1.63 | 3.43 | 1.57 | 0.76 |  | -1.45 | 3.36 | 1.03 | 0.74 |  |
|  |  | 2M3S | -1.91 | 3.8 | 1.22 | 0.73 |  | -1.98 | 3.7 | 0.88 | 0.77 |  |
|  |  | 2M4S | -1.92 | 3.79 | 1.27 | 0.74 |  | -2.2 | 4.04 | 0.79 | 0.79 |  |
|  |  | 2M5S | -2.07 | 3.9 | 1.36 | 0.76 |  | -2.07 | 3.79 | 0.95 | 0.74 |  |

Note: SS represents sole soybean with a density of 225,000 plants ha^-1^at Baotou and Linying site, SM represents single maize with a density of 75,000 plants ha^-1^ at Baotou site and 67,500 plants ha^-1^ at Linying site, 2M3S represents two rows of maize alternated with three soybean rows with a maize density of 75,000 plants ha^-1^ at Baotou site and 67,500 plants ha^-1^ at Linying site, 2M4S represents two rows of maize alternated with four soybean rows with a maize density of 75,000 plants ha^-1^ at Baotou site and 67,500 plants ha^-1^ at Linying site, 2M5S represents two rows of maize alternated with five soybean rows with a maize density of 75,000 plants ha^-1^ at Baotou site and 67,500 plants ha^-1^ at Linying site. a, b and c are model parameters, respectively, and R^2^ is the model coefficient of determination.
